# Taste Masked Microspheres of Ofloxacin: Formulation and Evaluation of Orodispersible Tablets

**DOI:** 10.3797/scipharm.1104-11

**Published:** 2011-07-25

**Authors:** Karan Malik, Gurpreet Arora, Inderbir Singh

**Affiliations:** 1 School of Pharmaceutical Sciences, Chitkara University, Solan-174103, Himachal Pradesh, India; 2 Chitkara College of Pharmacy, Chitkara University, Patiala-Chandigarh Highway, Rajpura-140401, Patiala, Punjab, India

**Keywords:** Microspheres, Solvent evaporation, Ofloxacin, Locust bean gum, Orodispersible tablets

## Abstract

Ofloxacin is a synthetic chemotherapeutic antibiotic used for treatment of a variety of bacterial infections, but therapy suffers from low patients’ compliance due to its unpleasant taste. This study was aimed to develop taste masked microspheres of ofloxacin using Eudragit and to prepare orodispersible tablets of the formulated microspheres using natural superdisintegrant. Taste masking Eudragit E100 microspheres were prepared by solvent evaporation technique with an entrapment efficiency ranging from 69.54 ± 1.98 to 86.52 ± 2.25%. DSC revealed no interaction between the drug and polymer. Microspheres prepared at a drug/polymer ratio of 1:4 and 1:5 revealed sufficient flow properties and better taste masking as compared to other ratios. Drug loaded microspheres were formulated as orodispersible tablets using locust bean gum as a natural superdisintegrant offering the advatages of biocompatibility and biodegrad-ability. The wetting time, water absorption ratio and in-vitro disintegration time of the tablets were found to range between 19 ± 2 to 10 ± 3 seconds, 59.11 ± 0.65 to 85.76 ± 0.96 and 22 ± 2 to 10 ± 2 seconds, respectively. The in-vitro ofloxacin release was about 97.25% within 2h. The results obtained from the study suggested the use of eudragit polymer for preparing ofloxacin loaded microspheres with an aim to mask the bitter taste of the drug and furthermore orodispersible tablets could be formulated using locust bean gum as a natural superdisintegrant.

## Introduction

Any medication which imparts an unpleasant taste is likely to result in poor patient compliance to a drug regimen especially for the children and elderly. Taste is a sensory response resulting from a chemical stimulation of taste buds on the tongue. Taste transduction is the process through which sense of taste is directed to the brain [1]. In general four different types of taste are there including sweet, sour, bitter and salty corresponding to which four different types of taste receptors are there. Sweet taste is mostly detected at the tip of the tongue, bitter taste at the back and the salty taste is sensed at the sides of the tongue [2]. Taste is detected when the soluble substances react with taste receptors and the perception of taste is transmitted to the brain via ninth cranial nerve. Various techniques to eliminate bitter taste of drugs include use of flavours [3], polymeric coatings [4], complexation with ion exchange resins [5], complexation with cyclodextrins [6], microencapsulation [7], chemical modifications etc. Microencapsulation is a process in which very thin coatings are applied around particles (solid/liquid). Therefore a microsphere is a small sphere having uniform coating around it, which provides it many useful properties like taste masking, controlled release etc. There diameter usually falls in a range of 1 μm to 1000 μm. Microspheres can be prepared by variety of methods including air suspension, coacervation phase separation, spray drying and congealing, pan coating, solvent evaporation and multiorifice centrifugation techniques [8]. Micro particulated entrapment of bitter drugs in the matrix of pH sensitive reverse enteric coated polymer, without compromising dissolution and bioavailability of drugs, were prepared by different methods has been reported in US Patent 20050136114 [9]. Eudragit E100 is a polymethacrylate with pH dependent solubility, specifically used for taste masking. It is soluble in gastric fluid up to pH 5.0 and is swellable and permeable above pH 5.0 [10]. So when the microencapsulated drug is consumed, it does not interact with taste receptors as it is insoluble in mouth. The moment the microencapsulated drug reaches the stomach the acidic pH conditions favour dissolution and thus the drug is released. Thus microencapsulation is a useful technique for masking the unpleasant taste. The most convenient method for preparation of microspheres is solvent evaporation technique. This technique is easier to perform in laboratory conditions [11]. Over other techniques it offers advantages like it does not use elevated temperatures like spray drying method thus suitable for thermo labile compounds. Control of particle size is easier in solvent evaporation method as compared to other methods.

The oral route of drug delivery is the most preferred route of administration of drugs for systemic action. Tablets are most widely used oral dosage forms as it provides advantages like ease of administration, transportability, compactness, non-invasive, easy to manufacture and contains a single accurate dosage of drug. In case of conventional tablets bed ridden, children and old age patients often face swallowing problems leading to poor patient compliance. To overcome these problems fast disintegrating tablets also known as orodispersible tablets are prepared which when placed on tongue disintegrates within seconds and the drug dissolves or get dispersed in saliva [12]. As no water is needed to administer these tablets, these offer an advantage of any time medicine for travellers who don’t have access to water. The technologies used for preparation of orodispersible tablets include lyophilization [13], moulding [14], direct compression [15], cotton candy process [16], spray drying [17], sublimation [18] and nanonization [19]. These techniques are based on the principles of increasing porosity and/or addition of superdisintegrants and water soluble excipients in the tablets [20].

Plant products nowadays are widely used as a substitute to synthetic products due to ease of local availability, lower prices as compared to synthetic products, biocompatible, biodegradable and environment friendly nature. Locust bean gum also called as carob bean gum is a galactomannan vegetable gum extracted from the seeds of the Carob tree (Ceretonia siliqua), mostly found in the Mediterranean regions. Locust bean gum has been widely used in food industry as a thickening and gelling agent [21]. Recently it has been established as a potential superdisintegrant in orodispersible tablets [22]. Owing to extensive swelling properties and superdisintegrant action of locust bean gum, it was used as superdisintegrant in orodispersible tablets containing taste masked microspheres of ofloxacin.

Ofloxacin is a synthetic fluorinated carboxyquinolone that has a broad spectrum of activity against both gram-negative and gram-positive bacteria [23]. It acts on DNA gyrase, an enzyme which like human topoisomerase, prevents the supercoiling of DNA during replication or transcription. Chemically, ofloxacin is a racemate, (±)-9-fluoro-3-methyl-10-(4-methylpiperazin-1-yl)-7-oxo-2,3-dihydro-7*H*-[1,4]oxazino[2,3,4-*ij*]quinoline-6-carboxylic acid. The usual dose of ofloxacin tablets is 200 mg to 400 mg orally every 12 h. The purpose of the present study was to formulate orodispersible tablets containing taste masked microspheres of ofloxacin by utilising a natural superdisintegrant (locust bean gum). The orodispersible tablets were evaluated for evaluated for parametric tests (thickness, diameter, hardness, tensile strength and friability), wetting time, water absorption ratio, effective pore radius, porosity, packing fraction, moisture uptake studies, in-vitro and in-vivo disintegration time, in-vitro release and stability studies.

## Experimental

### Materials

Ofloxacin was received as gift samples from Park Pharmaceuticals, Baddi, India. Eudragit E-100 was obtained as gift sample from Panacea Biotec, Lalru, India. Locust bean gum was obtained as gift sample from Lucid Colloids, Mumbai, India. Avicel PH-101(Batch No: RB13764) was procured from Sigma Aldrich, USA. Talc (Batch No: G635309) and magnesium stearate (Batch No: LB205509) were purchased from Loba Chemie, Mumbai, India. Hydrochloric acid (Batch No: 88893) and n-Hexane (Batch No: LB252809) were obtained from Thomas Baker and Loba Chemie, Mumbai, India respectively. All other chemicals and reagents were of analytical grade and were used as such.

### Preparation of Microspheres

Microspheres were prepared using solvent evaporation method. Firstly, Eudragit E100 was dissolved in mixture of organic solvent, ethanol: dichloromethane (8:2) on a magnetic stirrer (Eltek Instruments, MS 2012, India) to obtain uniform mixing. To this mixture, magnesium stearate (5% w/v) was added which served as droplet stabilizer followed by addition of ofloxacin with continuous stirring. The drug-polymer ratio was taken as 1:1, 1:2, 1:4 and 1:5. The polymer drug solution was added dropwise into a mixture of light liquid paraffin and n-Hexane containing Span 80 (2% w/v) maintained at 40 ºC. After complete evaporation of the solvent the microspheres were allowed to settle, supernatant was decanted and the microspheres were recovered by filtration through a Whatman filter paper (No. 41) followed by washing thoroughly with n-Hexane. The microspheres were dried at room temperature in a desiccator and stored in desiccator till further use.

### Evaluation of microspheres

#### Percentage yield determination

The prepared microspheres were completely dried in an oven maintained at 37°C for 24 h and then weighed. The percentage yield was calculated by the following formula:

$$\%\text{ Yield }=\;\frac{\text{Weight of microspheres}}{\text{Total weight of solid material}}\times 100$$

#### Entrapment efficiency

The entrapment efficiency was calculated by the formula

$$\text{EE }=\;\frac{\text{Practical drug content}}{\text{Theoretical drug content}}\times 100$$

Entrapment efficiency was calculated by digesting outer layer of 20 mg microspheres in 10 ml Chloroform and then 100 ml 0.1 N HCl was added. The suspension was then warmed for a few minutes, filtered & 1 ml of filtrate was made up to 10 ml with 0.1 N HCl. The solution was analysed using UV spectrophotometer (Systronics 2202) at 294 nm to determine amount of ofloxacin entrapped in microspheres. The calculations were made in triplicate.

#### Evaluation of flow properties of microspheres

The prepared microspheres were evaluated for flow properties including bulk density, tapped density, angle of repose, carr’s compressibility index and hausner ratio.

#### Particle size evaluation

Size distribution and average particle size of microspheres was calculated with optical microscopy. Optical microscope was fitted with eye piece micrometer which was then calibrated with a stage micrometer. Size of about 100 microspheres was calculated from each batch and then the average size was calculated.

#### Morphology

The morphology of the prepared batches of microspheres was evaluated by scanning electron microscopy (Hitachi S-520). Samples were mounted on aluminium stubs and coated with gold using a vacuum evaporator. Samples were then examined with a SEM microscope at an accelerating voltage of 10 kV.

### Compatibility studies

Chemical interaction between the drug and the polymeric material, if any, during the preparation of the microspheres was studied by Differential Scanning Calorimetry (DSC). DSC was carried out with the help of DSC equipment (DSC821e Mettler Toledo) calibrated using indium as a standard with a melting point (*T*_fus_) of 156.63°C and a calibration energy *Δ*_fus_ of 28.89 J/g. The temperature was increased from 25°C to 300°C at a heating rate of 10°C/min under a flow of nitrogen (80 ml/min).

### In-vitro taste evaluation of microspheres

Microspheres equivalent to 100 mg ofloxacin were placed in volumetric flask with 25ml of pH 6.8 phosphate buffer (simulated saliva) and were stirred for 5 min. the resulting mixture was filtered and analysed using UV-Visible spectrophotometer (Systronics 2202) at 294 nm.

### In-vivo taste evaluation of microspheres

A protocol for conducting taste assessment by taste panel studies was accepted by the Institutional Ethical Committee of Chitkara University (CU/CCP/MPh/2011/07). The prepared microspheres were subjected to taste evaluation test in 5 healthy male volunteers. All the volunteers were given details about the purpose, any risk involved and the procedure for taste evaluation. Taste evaluation of ofloxacin microspheres was conducted and the degree of bitterness was judged as per the five point scale given in table 1.

### Characterization of locust bean gum

Locust bean gum was characterized for swelling index, loss on drying (LOD), viscosity, pH and for microbial load. Microbial load was determined as outlined in Indian Pharmacopoeia 2007 for total aerobic count using plate count method. Pre-treated sample was inoculated on nutrient agar plates and were incubated for 96 hours and 120 hours at 34 ± 0.5 °C and 22 ± 0.5 °C for bacteria and fungi respectively. Then the number of colony forming units was calculated for bacteria and fungi.

### Preparation of tablets

Orodispersible tablets of ofloxacin microspheres were prepared using direct compression technique and locust bean gum as a superdisintegrant. The selected batches of microspheres were incorporated into tablet formulation. Criteria for selection of microspheres included there particle size, flow properties, encapsulation efficiency and taste masking ability. Microspheres equivalent to 100 mg ofloxacin were taken for each tablet composition (table 2). Avicel PH 101 was used as a directly compressible diluent. The directly compressible mixture were compressed using mutipunch tableting machine (AK Industries, India) fitted with 12.50 mm flat faced punch and die set possessing 50 ton compression force.

### Evaluation of tablets

#### Diameter and thickness

A calibrated vernier calliper (Indian calliper industries, Ambala, India) was used to evaluate diameter and thickness of tablets.

#### Hardness

The hardness of the tablets was determined by using Monsanto hardness tester (Pharma Chem Machineries, Mumbai, India). A tablet hardness of about 4–5 kg/cm^2^ is considered adequate for mechanical stability.

#### Friability

As per USP 30-NF 25, twenty six tablets were taken which corresponded to 6.5 g weight. The tablets were placed in a Roche friabilator (EI Products, India) and were rotated at 25 rpm for 4 minutes. The tablets were taken out, dedusted and reweighed. The percentage friability of the tablets was calculated by the formula,

$$\text{Percentage friability }=\frac{\text{Initial weight }-\text{Final weight }}{\text{Initial weight}}\times 100$$

#### Tablet tensile strength

The tablet tensile strength is the force required to break a tablet by compressing it in the radial direction. It is measured using a vernier calliper and monsanto hardness tester. For measuring the hardness of the tablets, the plunger of the hardness tester is driven down at a speed of 20 mm/min. Tensile strength for crushing (T) is calculated using equation:

$$\text{T }=\;\frac{2\text{F}}{\Pi \text{dt}}\;\times \;100$$

Where F is the crushing load, and d and t signify the diameter and thickness of the tablet, respectively.

#### Weight variation test

20 tablets from each batch were subjected to weight variation test. As per Indian Pharmacopoeia standards, the tablets should be within the specified limits i.e. ± 5% of average weight.

#### Wetting Time

A piece of tissue paper (10.75×12 mm) folded twice was placed in a culture dish (d=6.5 cm) containing 6 ml of simulated saliva (phosphate buffer pH 6.8). A tablet was carefully placed on the surface of tissue paper and the time required for simulated saliva to reach the upper surface of the tablet was noted as the wetting time [24].

#### Water Absorption Ratio

Test was done with the same procedure as that of wetting time. In this test initial weight of tablet was noted before placing on petri dish. After complete wetting the wetted tablet was then weighed. Water absorption ratio, R was determined using the equation,

$$\text{R }=\;\frac{W_{a}-W_{b}}{W_{b}}\times 100$$

Where, W_a_ is weight of tablet after water absorption and W_b_ is weight of tablet before absorption [24].

#### Effective pore radius (R_eff.P_)

R_eff.P_ of the powder blend was evaluated by method reported by Rana et al [25]. In this method a micropipette tip (2ml, transparent) was completely filled with powder and weighed (W_i_). Then n-hexane (surface tension (γ) 18.4 mN/m) was poured dropwise on bedtop till the solvent filtered out at the bottom of the tip. The tip was reweighed (W_f_). Effective pore radius was the calculated from the following formula. The experiments were repeated 3 times.

$$R_{\text{eff.P}}\;=\;\frac{W_{f}-W_{i}}{2\Pi \gamma }\times 100$$

#### Porosity

Porosity is a measure of the void spaces in a material, and is a fraction of the volume of voids over the total volume, between 0–1, or as a percentage between 0–100.

The porosity of the tablets was calculated as follows:

$$\varepsilon \;=1-\frac{m}{\rho_{\text{true}}\times V}$$

Where ρ true is the true density of the mixture, m and V are the weight and volume of the tablet, respectively. The true density of the powder was found using true density meter (SMART PYCNO 30, Maharashtra, India). In order to calculate true density, two pressure readings were taken. Initially helium gas was pressurised in a known reference volume and this reading was taken as first pressure reading. Then the gas is allowed to pass to sample cell containing the sample material due to which there is a drop in pressure as compared to initial pressure and this dropped pressure is taken as second pressure reading. Then material volume is calculated from which true density is calculated. When Helium is used initially vacuum is necessary to remove air from the pores of the sample. After that purging with Helium gas is done. Then the normal procedure is followed.

#### Tablet packing fraction

The tablet packing fraction (P_f_) is a measure of the degree of consolidation or compactness of the tablet. Tablet packing fraction was determined by:

$$\text{Packing fraction }{(P}_{f})\;=\;\frac{W}{\Pi r^{2}t\rho }$$

Where w is the weight of a tablet, r is radius, t is thickness and ρ is the particle density.

Ten tablets were used in each measurement. The radius and thickness of tablets were measured using a vernier calliper. The apparent particle density of the drug powder was determined using liquid paraffin displacement method. Firstly, the weight of a specific gravity bottle filled with liquid paraffin and the weight of the specific gravity bottle containing a sample of the drug powder (1000 mg) was noted and then it was filled with liquid paraffin after which the final weight was determined. The determination was performed in triplicate, and mean results were used in the calculation of P_f_. If the packing fraction is very high, fluid is unable to penetrate in the tablet which leads to slower disintegration [26].

#### In-vitro disintegration time

Disintegration time for orodispersible tablets was determined using USP disintegration apparatus (EI Products, Panchkula, India) with 0.1 N Hcl (900 ml at 37±1 °C) as the disintegrating medium. To comply with the test all of the tablets should disintegrate within 3 minutes as per official requirements as given in European Pharmacopoeia 2001.

#### In-vivo disintegration time [25]

A protocol for conducting disintegration time assessment was accepted by the Institutional Ethical Committee of Chitkara University. In vivo disintegration time was judged in five healthy male volunteers for each batch of tablets. The volunteers were previously well-versed for purpose of the study. Prior to the test the volunteers were instructed to rinse their oral cavity with distilled water. Each volunteer was asked to place one tablet on the tongue. Volunteers were strictly told not to chew or swallow the tablets, though licking was allowed. The end point for disintegration was taken when there was no lump left in the oral cavity. After the test was finished, volunteers were told to rinse there mouth properly.

#### Moisture uptake studies

Due to high content of hydrophilic excipients, orodispersible tablets have increased chance of moisture uptake which greatly affects stability of moisture sensitive products, so there is a need for special attention towards storage and packaging of orodispersible tablets. Therefore, moisture uptake studies are strongly recommended for orodispersible tablets [27]. The test was performed by keeping ten tablets in a desiccator (containing calcium chloride) for 24 hours at 37 °C to assure complete drying. The tablets were then weighed and stored for 2 weeks at 75% humidity. To achieve this humidity conditions, a saturated solution of sodium chloride was kept at the bottom of the desiccator for three days. On the tenth day tablets were re-weighed and the percentage increase in tablet weight was recorded.

#### In-vitro dissolution Studies

In vitro drug release of the prepared batches was determined using eight stage USP dissolution apparatus II (Lab India, DS 8000, Mumbai, India). The dissolution test was performed using 900 ml of 0.1 N Hcl buffer at 37 ± 0.5 ºC. The speed of rotation of paddle was set at 50 rpm. At a predetermined time interval, 5 ml samples were withdrawn, filtered through whatman filter paper, amply diluted and analysed at absorption maxima of 294nm according to Indian Pharmacopoeia 2007 using UV-Visible spectrophotometer (Systronics 2202, Ahmedabad, India). The in-vitro dissolution studies were performed in triplicate.

#### Stability testing

The prepared batches were evaluated for stability studies. During the full duration of study temperature and relative humidity of about 40 ± 2 °C and 75% RH respectively were maintained. The formulations were analysed at 0 day, 1 and 3 month time interval for hardness, friability, taste evaluation score, drug content and in-vitro disintegration time.

## Results and Discussions

### Evaluation of microspheres

Microspheres with different drug to polymer ratios were prepared using solvent evaporation technique. The prime parameters for selection of batches of microspheres for tableting were based on particle size, entrapment efficiency, flow characters and taste masking evaluation.

#### Percentage yield and Entrapment Efficiency

Percentage yield and entrapment efficiency were determined for the prepared batches of taste masked ofloxacin microspheres (table 3). Percentage yield obtained varied from 88.61 ± 0.52 to 96.33 ± 0.45. Entrapment efficiency increased with increase in polymer ratio but decreased with increase in stirring speed as the size of the microspheres reduced which resulted in decrease in entrapment efficiency (table 3).

#### Evaluation of flow properties

The prepared batches of microspheres were evaluated for bulk density, tapped density, angle of repose, carr’s consolidation index and hausner ratio. The results of powder flow properties (table 4) clearly indicate good flow characteristics for batches having drug to polymer ratio 1:4 and 1:5.

#### Particle size evaluation

**Fig. 1.**
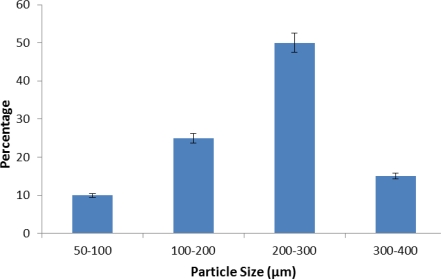
Histogram depicting particle size distribution of prepared microspheres at 400 rpm.

**Fig. 2.**
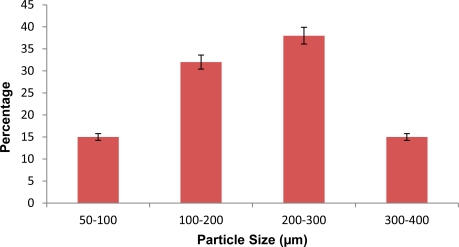
Histogram depicting particle size distribution of prepared microspheres at 800 rpm.

**Fig. 3.**
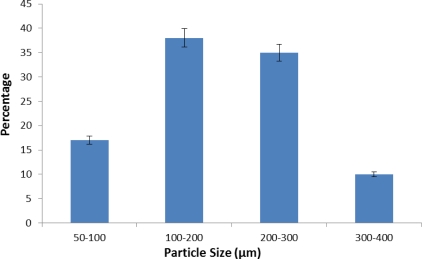
Histogram depicting particle size distribution of prepared microspheres at 1200 rpm.

Size distribution and average particle size of microspheres was calculated with optical microscopy. Upon increase in stirring speed there was reduction in average particle size of microspheres. The mean diameter of the prepared batches of microspheres (C1-C3) was found to be 270.52 μm, 232.23 μm and 205.54 μm respectively. Particle size distribution of microspheres is represented by various histograms (Figures 1–3).

#### Morphology

**Fig. 4.**
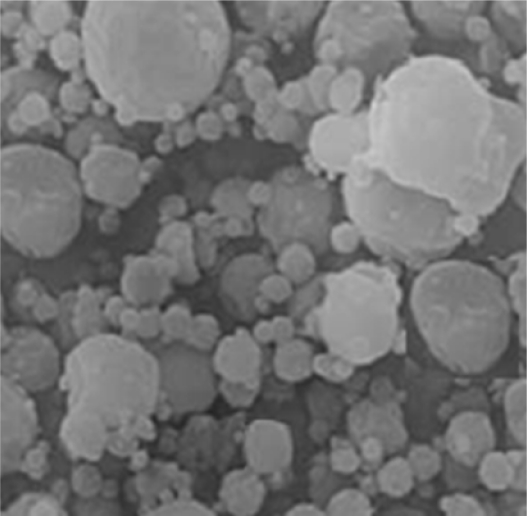
Scanning electron micrograph of microspheres (X100) having drug to polymer concentration 1:1

**Fig. 5.**
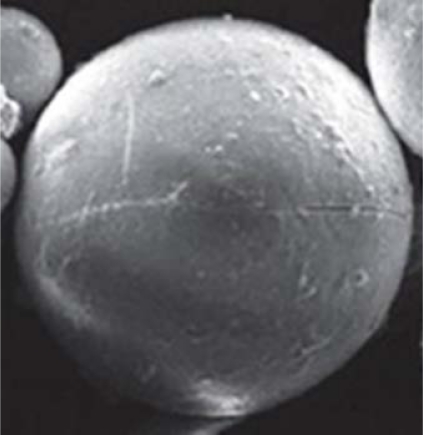
Scanning electron micrograph of microspheres (X1000) having drug to polymer concentration 1:4

The morphology of the prepared batches of microspheres was evaluated by scanning electron microscopy. Scanning electron micrographs are shown in figure 4 and 5. The micrographs of microspheres having drug and polymer ratio as 1:1 showed uneven surface and that of 1:4 showed to have smooth surfaces and bigger size. So, polymer concentration along with stirring speed was required to be controlled to an optimum level.

#### Chemical Interaction studies

**Fig. 6.**
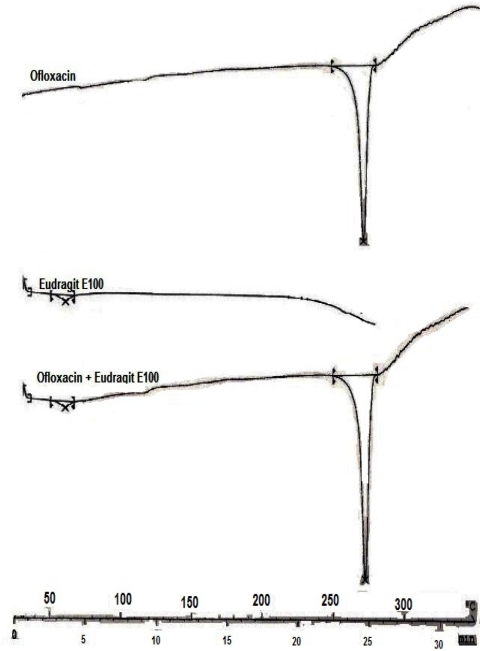
DSC Thermograms of Ofloxacin, Eudragit E100 and microspheres

In order to check chemical interaction between drug and polymer, thermal analysis was carried out by using Differential Scanning Calorimetry. DSC thermograms of Ofloxacin, Eudragit E100 and the formulation showed that there were no changes in the endotherms (Figure 6). The drug exhibited a sharp melting endotherm in the core and coated formulation.

#### In-vitro taste evaluation of microspheres

For evaluation of taste masking, drug release was studied in pH 6.8 phosphate buffer (simulated saliva). Microspheres with drug to polymer ratio 1:1 exhibited poorest taste masking and that of 1:4 and 1:5 showed excellent results for in-vitro taste masking.

#### In-vivo taste evaluation of microspheres

The prepared microspheres were subjected to taste evaluation test in 5 healthy male volunteers according to set protocol. The results of taste evaluation studies are shown in table 3. The microspheres having drug to polymer ratio 1:4 and 1:5 were found to be tasteless to most of the volunteers.

Based upon % yield, entrapment efficiency, flow properties, morphology and taste masking properties, batch C1 was found to be optimum and was further selected for tableting.

#### Characterization of locust bean gum

Swelling index of 1% w/v solution of locust bean gum was found to be 2000 which indicated toward good swelling tendency of the natural gum. Loss on drying was obtained to be 13.53% which was well within limits for locust bean gum (max 14%). Viscosity of 1% w/v solution of locust bean gum using spindle number 62 of Brookfield viscometer at 37 ± 1 °C was found to be 83, 81, 82.5, 80.4 and 78 at 5, 10, 20, 50 and 100 rpm respectively. pH of 1% w/v solution at 37 ± 1 °C was found to be 5.79.

As natural materials are prone to possess microbial contamination so it becomes necessary to perform microbial load studies. Total aerobic count using plate count method was determined as given in Indian Pharmacopoeia 2007. The number of colony forming units were found to be 99 CFUs/g and 13 CFUs/g for bacteria and fungi respectively which were well within limits as per Indian Pharmacopoeia 2007 for total aerobic count.

#### Tablet evaluation

**Fig. 7.**
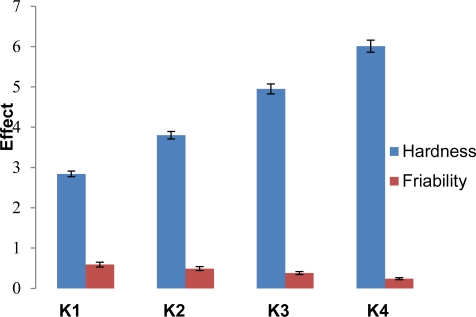
Effect of locust bean concentration on tablet hardness and friability.

**Fig. 8.**
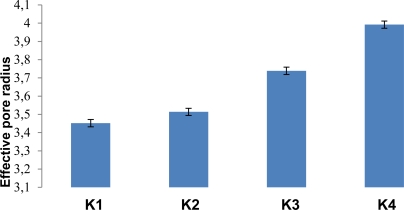
Effect of locust bean gum on effective pore radius

**Fig. 9.**
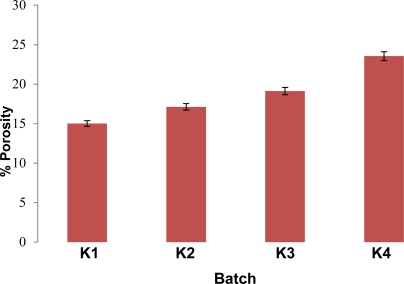
Effect of locust bean gum on tablet porosity.

All the batches of orodispersible tablets were formulated under similar conditions to avoid processing variables. The tablets prepared by direct compression method were found to be free from capping, chipping and sticking. The prepared tablets were evaluated for various physical parametric tests (table 5). An appreciable effect was seen on tablet hardness, friability and tensile strength due to increasing concentration of locust bean gum (figure 7). All of the batches passes weight variation test. Wetting time, water absorption ratio and in-vitro disintegration time were found to be ranging between 19 ± 2 to 10 ± 3 seconds, 59.11 ± 0.65 to 85.76 ± 0.96 and 22 ± 2 to 10 ± 2 second respectively. R_eff.P_ is an indicator of tablet porosity. R_eff.P_ and porosity (figure 8 and 9) were found to be ranging from 3.452 ± 0.15 to 3.992 ± 0.86 mm and 15.006% to 23.547 % respectively indicating appreciable capability of locust bean gum to increase water penetration due to wicking action which increases porosity thus lowers disintegration time with increase in polymer concentration. The tablet packing fraction (P_f_) is a measure of the degree of consolidation or compactness of the tablet. Tablet packing fraction was found to be 0.843, 0.822, 0.802 and 0.764 (K1–K4) which indicated towards good porosity imparting character of the natural polymer. The in-vivo disintegration time was found to be 22 ± 2 to 10 ± 2 seconds (K1–K4). In vivo performance of the formulated orodispersible tablets using locust bean gum as superdisintegrant was well in line with the in vitro results.

**Fig. 10.**
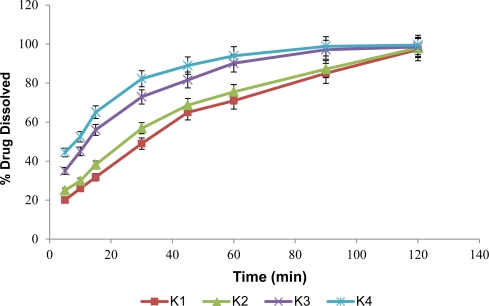
Dissolution profile of prepared orodispersible tablets containing taste masked ofloxacin microspheres.

In vitro ofloxacin release was 97.25% (K1), 99.55% (K4) for the prepared batches of orodispersible tablets (figure 10).

Stability studies for the prepared batches containing locust bean gum as superdisintegrant was performed which indicated that there was no significant change in tablet hardness, friability, taste evaluation score, drug content and in-vitro disintegration time (table 6).

## Conclusions

The purpose of the present study was to develop taste masked microspheres of ofloxacin using Eudragit E100 employing solvent evaporation technique as method of preparation of microspheres. The formulated microspheres were incorporated into tablet dosage form using locust bean gum as natural tablet superdisintegrant. In conclusion eudragit polymers have excellent taste masking potential and natural excipients could prospectively be used as nutraceuticals for their pharmaceutical applications.

## Figures and Tables

**Tab. 1.** Five point scale for taste evaluation

**Taste Characteristics**	**Score**
Pleasant	0
Tasteless	1
Slightly bitter	2
Moderately bitter	3
Intensely bitter	4

**Tab. 2.** Composition of orodispersible tablet formulations

**Ingredients (mg)**	**K1**	**K2**	**K3**	**K4**
Microspheres containing Ofloxacin equivalent to	100	100	100	100
Locust bean gum	15	30	45	60
Avicel PH 101	73	58	43	28
Talc	6	6	6	6
Magnesium stearate	6	6	6	6
Total weight	600	600	600	600

**Tab. 3.** Microsphere evaluation parameters

**Batch**	**Ofloxacin (Drug) (in mg)**	**Eudragit (Polymer) (in mg)**	**D:P Ratio**	**Stirring Speed**	% Yield ± S.D.*	Entrapment Efficiency (%) ± S.D.^*^	**Taste masking score**
A1	100	100	1:1	400	89.52 ± 0.52	73.41 ± 1.26	3
A2	100	100	1:1	800	92.41 ± 0.26	72.56 ± 2.51	3
A3	100	100	1:1	1200	93.64 ± 0.45	69.54 ± 1.98	3
B1	100	200	1:2	400	91.52 ± 0.78	81.25 ± 1.58	2
B2	100	200	1:2	800	88.61	80.52 ± 1.38	2
B3	100	200	1:2	1200	92.44	78.93 ± 1.14	2
C1	100	400	1:4	400	97.21 ± 0.41	84.93 ± 1.65	1
C2	100	400	1:4	800	94.56	83.33 ± 2.75	1
C3	100	400	1:4	1200	92.06	80.01 ± 2.65	1
D1	100	500	1:5	400	96.54 ± 0.45	86.52 ± 2.25	1
D2	100	500	1:5	800	95.42	85.97 ± 1.03	1
D3	100	500	1:5	1200	96.33 ± 0.54	84.93 ± 2.12	1

* Values are mean ± SD (n=3)

**Tab. 4.** Microsphere powder flow properties

**Parameters**	**Results**					
	**A1**	**A2**	**A3**	**B1**	**B2**	**B3**

Bulk Density(g/cm^3^)	0.60 ± 0.02	0.60 ± 0.15	0.58 ± 0.05	0.56 ± 0.12	0.58 ± 0.05	0.55 ± 0.09
Tapped density(g/cm^3^)	0.59 ± 0.12	0.58 ± 0.03	0.54 ± 0.04	0.50 ± 0.02	0.51 ± 0.08	0.45 ± 0.06
Carr’s compressibility index (%)	38.33 ± 0.55	36.66 ± 0.19	35.11 ± 0.22	33.28 ± 0.44	29.93 ± 0.67	26.81 ± 0.23
Angle of Repose	42.23 ± 0.11	40.52 ± 0.23	39.12± 0.25	37.56 ± 0.34	35.22 ± 0.45	31.21 ± 0.23
Hausner Ratio	1.01 ± 0.02	1.03 ± 0.01	1.07 ± 0.04	1.12 ± 0.02	1.13 ± 0.01	1.22 ± 0.05

**Parameters**	**Results**					
	**C1**	**C2**	**C3**	**D1**	**D2**	**D3**

Bulk Density(g/cm^3^)	0.58 ± 0.03	0.59 ± 0.01	0.61± 0.03	0.55 ± 0.11	0.59 ± 0.04	0.57 ± 0.04
Tapped density(g/cm^3^)	0.46 ± 0.05	0.47 ± 0.02	0.50 ± 0.01	0.43 ± 0.12	0.47 ± 0.22	0.45 ± 0.15
Carr’s compressibility index (%)	21.31 ± 0.21	20.66 ± 0.12	20.96 ± 0.23	23.18 ± 0.66	20.66 ± 0.57	21.94 ± 0.78
Angle of Repose	28.12 ± 0.56	28.05 ± 0.12	27.89 ± 0.23	27.56 ± 0.12	27.23 ± 0.67	27.05 ± 0.11
Hausner Ratio	1.26 ± 0.05	1.25 ± 0.01	1.22 ± 0.06	1.28 ± 0.02	1.25 ± 0.01	1.26 ± 0.02

**Tab. 5.** Evaluation of prepared tablets

**Parameters**	**K1**	**K2**	**K3**	**K4**
Diameter (mm)	12.50 ± 0.05	12.51 ± 0.03	12.50 ± 0.02	12.50 ± 0.04
Thickness (mm)	4.71 ± 0.03	4.75 ± 0.04	4.73 ± 0.02	4.78 ± 0.04
Friability (%)	0.59 ± 0.04	0.48 ± 0.05	0.38 ± 0.01	0.24 ± 0.03
Hardness (kg/cm^2^)	2.84 ± 0.54	3.80 ± 0.23	4.95 ± 0.55	6.01 ± 0.75
Tensile strength (MN/m^2^)	0.468 ± 0.15	0.684 ± 0.12	1.02 ± 0.05	1.198 ± 0.10
Weight variation test	Pass	Pass	Pass	Pass
Wetting time (sec)	19 ± 2	16 ± 1	14 ± 2	10 ± 3
Water absorption ratio %	59.11 ± 0.65	69.95 ± 0.84	76.45 ± 0.78	85.76 ± 0.96
In-vitro disintegr. time (sec)	20 ± 1	15 ± 2	14 ± 2	12 ± 1
In-vivo disintegr. time (sec)	22 ± 2	17 ± 3	13 ± 1	10 ± 2
Moisture uptake (%)	0.71 ± 0.13	0.75 ± 0.25	0.82 ± 0.19	0.84 ± 0.26

**Tab. 6.** Stability study data of orodispersible tablets

	**Parameter (Months)**								
	**Hardness**			**Friability**			**Taste evaluation score**		
	
**Batch**	**0**	**1**	**3**	**0**	**1**	**3**	**0**	**1**	**3**

K1	2.84 ± 0.54	2.71 ± 0.12	2.52 ± 0.80	0.59 ± 0.04	0.62 ± 0.09	0.65 ± 0.05	1	1	2
K2	3.80 ± 0.23	3.52 ± 0.30	3.27 ± 0.10	0.48 ± 0.05	0.52 ± 0.04	0.58 ± 0.12	1	1	2
K3	4.95 ± 0.55	4.55 ± 0.35	3.95 ± 0.22	0.38 ± 0.10	0.41 ± 0.05	0.43 ± 0.06	1	1	2
K4	6.01 ± 0.75	5.74 ± 0.34	5.52 ± 0.55	0.24 ± 0.01	0.27 ± 0.04	0.33 ± 0.05	1	1	2

	**Parameter (Months)**											
	**Drug content**			**In-vitro disintegration time**					
				
**Batch**	**0**	**1**	**3**	**0**	**1**	**3**			
			
K1	99.24 ± 0.25	98.50 ± 0.10	97.54 ± 0.25	20 ± 1	21 ± 2	23 ± 3			
K2	98.45 ± 0.66	98.44 ± 0.55	98.66 ± 0.85	15 ± 1	17 ± 2	19 ±1			
K3	98.98 ± 0.15	97.23 ± 0.50	96.56 ± 0.35	14 ± 2	15 ± 1	17 ± 2			
K4	97.12 ± 0.66	96.85 ± 0.33	96.52 ± 0.14	12 ± 2	13 ± 1	15 ± 1			
